# Identifying legitimate websites selling medicines in South Africa

**DOI:** 10.4102/hsag.v29i0.2732

**Published:** 2024-11-12

**Authors:** Divishka Balraj, Nomvelo Mntambo, Kuyabonelelwa M. Lembede, Menelisi Madikane, Della-Reece Daniel, Deanne Johnston

**Affiliations:** 1 Discipline of Pharmaceutical Sciences, College of Health Sciences, University of KwaZulu-Natal, Durban, South Africa

**Keywords:** Community pharmacy, pharmacy websites, illegal medicines, illegal websites, online medicines

## Abstract

**Background:**

The online sale of medicines has skyrocketed globally, where medicines are purchased in the comfort and privacy of one’s own home. However, there are an increasing number of illegal websites selling counterfeit and falsified medicines.

**Aim:**

The aim of this study was to review the information and medicines sold online through pharmacy websites in South Africa (SA) and their compliance with local legislation.

**Setting:**

The study setting comprised online pharmacy websites in SA.

**Methods:**

This quantitative descriptive study used a purposeful questionnaire based on the South African Pharmacy Council inspection questionnaire for pharmacies operating websites. Websites claiming to be an online pharmacy in SA were included.

**Results:**

There were 25 websites reviewed, which claimed to be online pharmacies. Majority (*n* = 22) were found to be legal websites, operated by a registered community pharmacy and required a prescription for the purchase of prescription only medicines. Few websites complied with legislation such as displaying the Y number (*n* = 5) and name of the responsible pharmacist (*n* = 10). The remaining three websites were not linked to physical pharmacies, none complied with legislation and supplied medicines without a prescription.

**Conclusion:**

Although the online sale of medicines is regulated in SA, not all websites complied with legislation. The study highlighted the importance of monitoring websites claiming to be online pharmacies and their compliance with legislation.

**Contribution:**

Four stakeholders, consumers; pharmacy websites; regulators and healthcare providers, were identified and their role outlined in promoting the safe online purchasing of medicines.

## Introduction

The sale of medicines on digital platforms, such as websites and mobile applications, has grown significantly and gives billions of people around the world access to a range of medicinal supplies at the click of a mouse. Although one may initially think an online pharmacy sells medicine to the public from a website, there are other services that may be provided such as the sale of health-related products, counselling on medicines and health-related advice as well as links to support groups (Gray 2011).

An online pharmacy offers anonymity where the consumer can conveniently purchase medication in the privacy of their own home searching the web for the best price (Jain, Tadv & Pawar 2017). However, there may also be negative implications such as the consumer being unable to inspect the medicine on purchase, and that they will most likely receive little to no consultation on the use of the medicine and possible interactions are most likely not going to be picked up especially if purchasing medicines from multiple sites (Jain et al. 2017).

The online sale of illegal medicines has risen considerably where consumers may be accessing counterfeit, falsified and/or inappropriate medicines (Jack 2016; Lee et al. 2017). An example of this was the sale of ivermectin during the coronavirus disease 2019 (COVID-19) pandemic when its sales soared. Fittler et al. (2021) found that there were far more illegitimate retailers selling ivermectin online and without a valid prescription. In a systematic review on the sale of prescription medicine from online pharmacies, they found that consumers resorted to illegal online pharmacies for the purchase of medicines such as opioids for which they struggled to obtain prescriptions (Long et al. 2022).

The online sale of medicine provides many more opportunities for the illegal sale and misuse of medicines, including counterfeit and falsified medicines; therefore, there is an urgent need for greater regulatory control for international organisations and statutory bodies. A study conducted by the International Pharmaceutical Federation (FIP) in 2021 found that half the countries surveyed (*n* = 79) did not have specific criteria, which regulated the online sale of medicines (Dineen-Griffin 2023).

**FIGURE 1:**
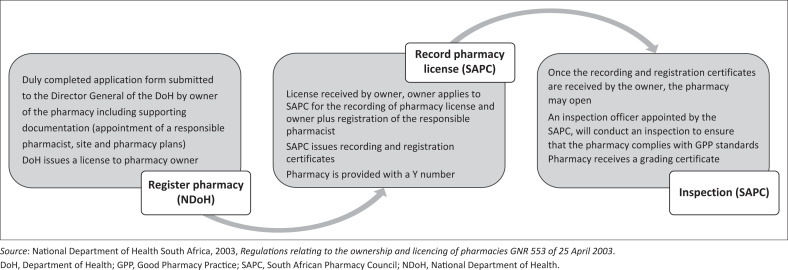
An overview of the process of registering and recording of the pharmacy by the pharmacy owner.

In South Africa however, the online sale of medicines is regulated, where medicines sold need to comply with regulations set out in the *Medicines and Related Substances Act 101 of 1965* (South Africa Government 1965). Prescription medicines can be purchased through websites linked to a community or institutional pharmacy licensed by the Department of Health as well as recorded with the South African Pharmacy Council (SAPC) (National Department of Health South Africa [NDoH] 2003). The process of opening a pharmacy in South Africa is outlined in Figure 1.

The South African Health Products Regulatory Authority (SAHPRA) is responsible for the registration of medicines in South Africa. Unregistered medicines may only be sold under specific circumstances (South Africa Government 1965). According to the *Medicines and Related Substances Act 101 of 1965* (as amended) (South Africa Government 1965), scheduled medicines, may only be sold to the public by a pharmacist and/or pharmacy support personnel, in accordance with their scope of practice and under the direct supervision of a pharmacist, from a community or institutional pharmacy with a license issued by the Department of Health and recorded with the SAPC. Veterinarians, medical practitioners, dentists, nurses and other registered persons are permitted to dispense medication to patients who have consulted them, provided they are a holder of a section 22C (1) (a) license issued by the Department of Health.

Pharmacies are required by law to provide high-quality pharmaceutical services that comply with Good Pharmacy Practice (GPP) standards as provided by the SAPC (South Africa Government 1974). The minimum standards for pharmacies, community and institutional, operating websites are defined in the GPP Manual and associated SAPC rules state that ‘A website that is used to sell medicines must be operated by a pharmacy’ (SAPC 2010). Therefore, the website is the responsibility of the associated pharmacist and needs to comply with the relevant legislation. Even though a patient will not physically visit the pharmacy, a pharmacist must be available for consultation. Furthermore, the process for handling prescriptions sent electronically is delineated, where the original prescription must be provided within seven working days. The standard outlines the requirements for a website where the specific information, such as the name of the responsible pharmacist and the Y number, must be found on the homepage as well as providing certain policies and procedures. Furthermore, an inspection officer, appointed by the SAPC, will review if websites operated by the community or hospital pharmacy comply with these criteria when conducting a physical inspection of the brick-and-mortar pharmacy.

To our knowledge, there has been no research conducted on websites operated by pharmacies in South Africa. Thus, the aim of this study was to review the information and schedules of medicines sold on pharmacy websites in South Africa as well as to investigate whether they meet the minimum requirements as stated in the GPP.

## Research methods and design

This descriptive quantitative study reviewed websites claiming to be an online pharmacy selling medicines in South Africa. Search engines, Google Chrome, Microsoft Edge and Firefox, were used to search for websites with the following key phrase ‘online pharmacy South Africa’.

A purposeful report form was designed based on the criteria for pharmacies providing services from a website, found within the inspection questionnaires for community and hospital pharmacies (SAPC n.d.). Dichotomous questions were used to assess if a website complies with the minimum standards set out in the GPP (SAPC 2010). The name, Y number, address and name of each responsible pharmacist was verified searching the register on the SAPC website.

### Data analysis

The data were captured on Microsoft Forms, checked by a co-investigator, and then extracted to Microsoft Excel. The data were assessed quantitatively making use of descriptive analyses as well as graphical representations and comparison tables.

### Ethical considerations

Given the information analysed was in the public realm, an ethics exemption was received for this study from the Biomedical Research Ethics Committee (BREC) of the University of KwaZulu-Natal (Ethics Number: 00022221).

## Results

The study examined the information provided by 25 pharmacy websites and determined if they complied with criteria outlined in the GPP (SAPC 2010). The 25 websites were divided into two groups based on whether they were linked to a physical pharmacy.

Of the 25 websites, 22 could be linked to registered physical community pharmacies. The physical pharmacies were verified by searching for the pharmacy name. These websites were referred to as ‘Legitimate pharmacy websites’.

The three remaining websites, could not be located in the SAPC register and there was no information referring to these websites linking them with registered pharmacies. Therefore, these websites were referred to as ‘Illegitimate pharmacy websites’.

### Legitimate pharmacy websites

It is important to notice that of the 22 websites that were linked to physical pharmacies, there were 10 websites that were each linked to one physical pharmacy and 12 websites linked to more than one physical pharmacy. The latter would most likely indicate that these pharmacies belonged to a group and the website was associated with the group as opposed to a single premises.

All 22 websites provided the physical addresses of the pharmacies. Websites linked to more than one physical pharmacy, provided the name and physical address of each pharmacy. This information was either listed on the webpage or could be found using a search function or drop-down list. In addition, the operating hours of all these pharmacies were found on the websites.

**FIGURE 2:**
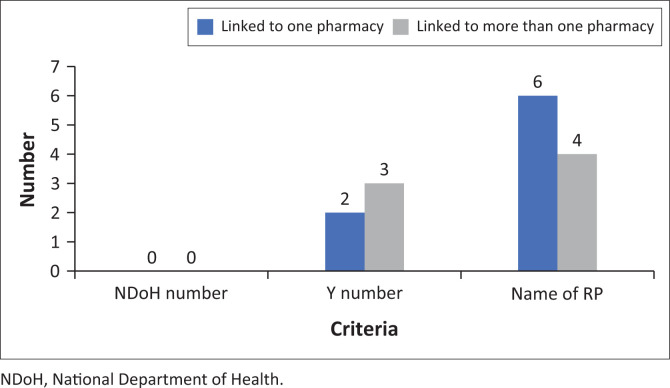
Pharmacy registration details displayed on websites.

There was only one pharmacy which stated on the website that the pharmacy was licensed by the NDoH and recorded with the SAPC. None of the websites provided the NDoH number and only five pharmacies provided the Y number of the physical pharmacy (Figure 2).

Less than half (*n* = 10) of the websites provided the name of the responsible pharmacist (Figure 2). Even fewer pharmacies (*n* = 4) referred to methods for direct communication with a pharmacist.

**FIGURE 3:**
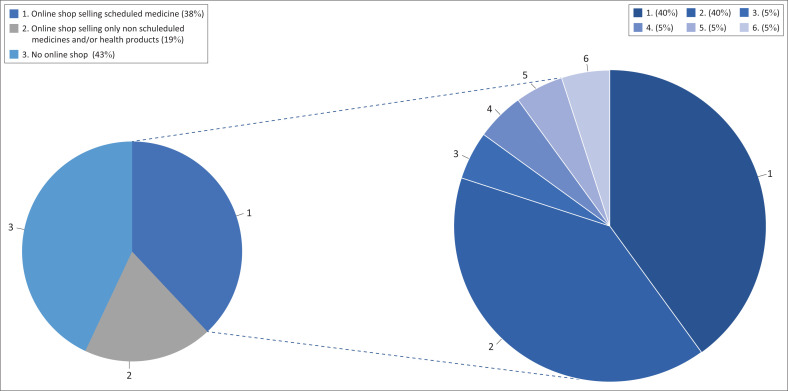
Online sale of medicines from websites operated by community pharmacies.

There were 11 websites that had an online shop where users were able to click on the product they required, which was added to the basket. A few websites (*n* = 4) sold only Schedule 0 and/or non-scheduled medicines, which do not require a prescription (Figure 3). The remaining seven websites sold Schedule 1 and 2 medicines in addition to Schedule 0 and/or non-scheduled medicines. No prescription was required by consumers purchasing these medicines (Figure 3).

Only one website allowed consumers to select Schedule 3–6 medicines from the website, which could be purchased provided the consumer had a valid prescription (Figure 3). In addition, only when purchasing a Schedule 6 medicine was the original prescription required to be posted or couriered to the pharmacy.

**FIGURE 4:**
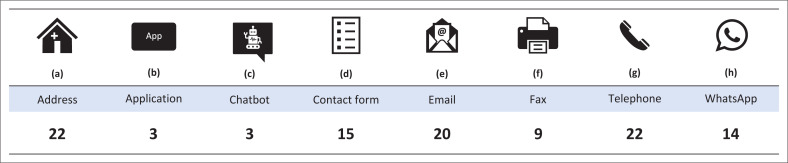
Various communications methods linked to the website.

The remaining websites (*n* = 21) requested the consumer to submit a copy of their prescription when ordering via the application, contact form, email, fax and/or WhatsApp (Figure 4). Once the pharmacy received the prescription, the pharmacy would communicate with the consumer. These websites did not state that the original prescription was required, other than when purchasing Schedule 6 medicines.

The pharmacy websites allowed the consumer to communicate via multiple methods. As required by the GPP, all websites provided their physical address and telephone number. The majority provided an email address (*n* = 20) while many sites directed consumers to use their contact form (*n* = 15) and/or using an instant messaging service such as WhatsApp (*n* = 14).

### Illegitimate websites

Three websites could not be linked to a physical pharmacy in South Africa although the uniform resource locator (URL) indicated that the website is hosted locally. They did not provide information regarding registration with the NDoH nor the SAPC. They also did not provide a physical address of a pharmacy, nor was the name of the responsible pharmacist given. Although these websites provided telephone numbers, they were international numbers. Other than providing a telephone number, the websites provided online chat support and consumers were given the option to complete an online contact form.

These websites referred to the medicines listed on their sites as being registered by international regulatory authorities or sold internationally. In addition, none of the websites refer to the medicines sold as being registered in South Africa. Although it was outside the scope of the project to determine if the medicines sold on the websites were registered for use in South Africa, it is worth noting that many brand names or some active pharmaceutical ingredients could not be verified on the SAHPRA website. In addition, the pictures provided on these sites were of the dosage form (e.g. a picture of the tablet) or primary packaging (blister pack).

None of these websites requested that a prescription be provided upon ordering, even if a medicine containing the same active pharmaceutical ingredient when sold in South Africa is registered as a Schedule 3 or above. Two of the websites stated that although a prescription is not required, they advise the customer to consult their doctor before purchasing the medicine.

## Discussion

This study reviewed 25 websites that claimed to be online pharmacies in South Africa, where majority (*n* = 22) were found to be legal websites and were operated by a registered community pharmacy. Although pharmacies operating websites in South Africa are regulated (SAPC 2010), it was found that these rules were only partially followed. The remaining three websites could not be linked to a registered brick-and-mortar pharmacy and were considered to be illegitimate.

Signs that consumers may look for in identifying legitimate websites are details that the website is operated by a licensed pharmacy and provides for access to a licensed pharmacist to answer their questions (Jain et al. 2017). Although it was possible to identify and verify that the legitimate pharmacy websites were linked to one or more pharmacies registered by NDoH and recorded with SAPC, only one website stated this. Adding to this none of the pharmacies provided a NDoH number and very few (*n* = 5) provided Y numbers. Provision of registration details is further complicated when a website is associated with a group of pharmacies as each pharmacy has their own registration details and legislation does not cater to these scenarios.

The study showed that websites defined as ‘illegitimate’ provided foreign phone numbers and did not provide a physical address. Fittler, Bősze and Botz (2013) warn that address and telephone numbers provided by online pharmacies are often not the same as that of the operator. This in turn would allow operators to sell medicines manufactured elsewhere in the world. Likewise, the three ‘illegitimate’ websites referred to medicines they sold as having been approved by international regulatory authorities.

It is important for consumers purchasing any medicine to be able to communicate with a pharmacist and for pharmacists to be in a position to provide advice on the safe use of medication. It is suggested that this is particularly important for self-medication after accessing online pharmacy websites (Dorokhova et al. 2023). It is therefore concerning that the majority of websites did not provide the name of the responsible pharmacist (*n* = 12) and less than 20% (*n* = 4) referred to direct communication with a pharmacist. In South Africa, Schedule 0 and 1 medicines may be advertised directly to the public; however, only the name, pack size, strength and price of Schedule 2 and above medicines can be displayed (General Regulation 42 1–3). As only limited information may be advertised on the websites, it is even more important for consumers to consult with a pharmacist.

The results from this study were in contrast to the study by Fittler et al. (2013), which found that more than 90% of their study sample did not request a medical prescription. All the websites in this study associated with a brick-and-mortar pharmacy requested the patient to provide a prescription when purchasing Schedule 3 and above medication. Consumers were further provided with various methods to upload or submit the prescription. However, the detail of submitting the original prescription was not explicitly explained on any of the websites. According to legislation, when a prescription is sent electronically, the original prescription must reach the pharmacy within seven working days (SAPC 2010). If the pharmacy does not receive the original prescription, the consumer may use the same prescription to purchase medication from another pharmacy. The use of advanced technology, such as electronic prescription services compliant with advanced electronic requirements, may have positive impact when purchasing medicines from online pharmacy websites, as this requires the prescription to be sent directly from the doctor to the pharmacy.

The websites defined here as illegitimate did not require a prescription to be submitted but shifted the responsibility to the consumer to consult with their doctor before taking medication. There is a far greater risk of patients receiving substandard and/or falsified medicine when purchasing medicines from unlicensed websites (World Health Organization 2018). Moreover, consumers have free access to purchase as much as they want and to use the medicine at their own discretion. Almomani, Patel and Donyai (2023) found that participants in their study who purchased medicines online wanted to evade the legal requirements to obtain prescription medication and were aware of the associated risks.

**FIGURE 5:**
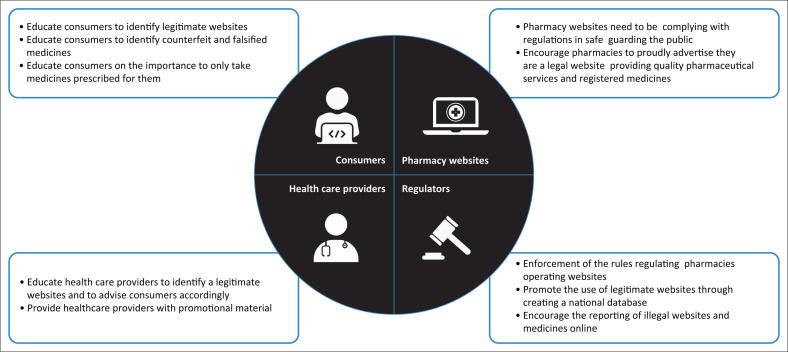
Four stakeholders groups identified in the online sale of medicines in South African consumers.

The results from the study indicate there is a need to ensure that the sale of medicines online is regulated and the regulations are adhered to. We propose that multiple partners are required in achieving this goal and four stakeholder groups were identified namely, consumers, pharmacy websites, regulators and healthcare professionals (Figure 5).

### Consumers

**FIGURE 6:**
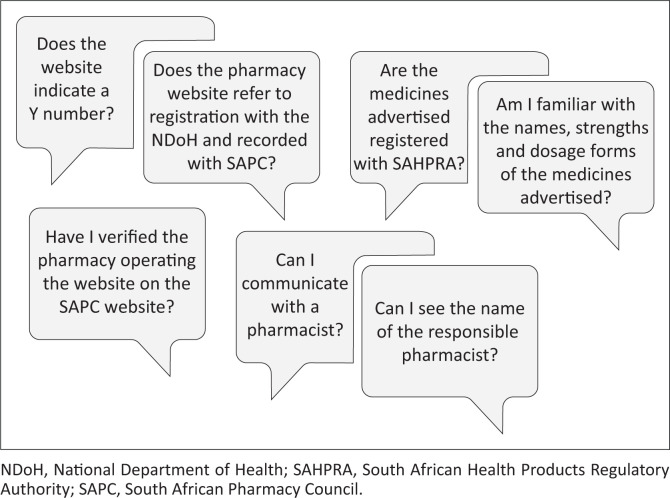
Questions consumers should ask when purchasing medicine online.

It is critically important that consumers are able to identify legitimate websites and are adequately informed and educated to safely purchase medicines online (Fincham 2021). Thus, in South Africa consumers should only use websites associated with physical community and hospital pharmacies registered by the NDoH and recorded by the SAPC, as well as can only purchase medication that is registered with SAHPRA. Consumers should be encouraged to ask themselves the questions in Figure 6.

Consumers need to be alerted that websites that do not require a prescription when purchasing prescription only medication (Schedule 3–6 medicines) are possibly illegal and medicines sold on these sites are more likely to be counterfeit or falsified.

### Owners of the pharmacy websites

This study indicated that relatively few websites complied with the minimum standards for pharmacies operating websites. Punitive measures have been put in place to ensure pharmacy websites display this information. One may suggest that a proactive stance should be taken, where websites should proudly advertise that they are a legal website and consumers using their site can rest assured they are receiving high-quality pharmaceutical services and registered medicines.

### Regulatory authorities

The duties of SAPC and SAHPRA are clearly demarcated. The SAPC, responsible for the pharmacy professionals, is chiefly concerned with the registration of persons and pharmacy premises (South Africa Government 1974). While SAHPRA regulates medicines, including their registration, monitoring and evaluation (South Africa Government 1965). Therefore, the online sale of medicines is the dual and combined responsibility of both regulators in controlling who can sell medicines online and what may be sold.

Compliance with the minimum standard for pharmacies operating websites is monitored by inspection officers when inspecting the brick-and-mortar pharmacies (SAPC 2010). However, non-compliance with the minimum standard does not automatically result in the pharmacy being given a poor grading, Grade C rating (SAPC 2020). Thus, it is questionable if the consequences for disregarding these specific rules are sufficient. It is proposed that the inspection of websites selling medicines is conducted independently of the monitoring inspection of the physical pharmacy.

Consumers are able to verify pharmacies on the SAPC websites and medicines on the SAHPRA websites through searching the applicable register. However, one may question how consumers would know where or what to search for without advertising this functionality. The following two examples of campaigns aimed at empowering consumers to identify registered pharmacy websites have been successfully implemented:

In the United States, the Food and Drug Administration (FDA) 2020 has run a campaign entitled ‘BeSafeRx: Your Source for Online Pharmacy Information’ which is aimed at consumers and assists them in locating a licensed pharmacy.

When purchasing medicines online in European Union member states, websites are required to register with their regulatory authority (Barroso 2014). Once registered, the website is required to display a common logo. When the consumer clicks on the logo, they will be directed to a website listing approved websites. The consumer is therefore encouraged through this facility to search only for legitimate pharmacy websites.

It is recommended that the regulators embark on an educational campaign to help South African consumers to navigate legitimate websites for purchasing of medicines online. In addition, the reporting of illegal websites and medicines by the public should be encouraged and the process outlined.

### Healthcare providers

A study dealing with the issue of online sales and purchasing found that Italian community pharmacists were not in support of the online sale of prescription and non-prescription medicines despite its regulation (Lombardo, Marino & Cosentino 2020). Another study from the United States found that pharmacists were unable to determine if the online pharmacy website met the legal requirements and were not confident in advising patients on whether the websites were illegal (Hertig et al. 2021). However, with the rapid proliferation of online pharmacies and their potential benefits to consumers, it is suggested that pharmacists should be encouraged to support advancements in technology while maintaining their professional role (Dineen-Griffin 2023).

Healthcare providers, in particular prescribers and pharmacists, have the responsibility to uphold the laws and regulations, which govern the prescription and sale of medicines (South African Government 1965). In the United States, the FDA has identified the role healthcare professionals play in supporting and educating consumers on the purchasing of medicines online (FDA 2020) and provides links to educational material for healthcare professionals to download to display and share with patients. Hence, it is recommended that further research is needed to determine the perceptions of South African healthcare providers as well as providing them with promotional material to aid the safe online purchasing of medicines.

## Strengths, limitations and areas for further research

### Strengths

To our knowledge, this study was the first study researching websites selling medicines in South Africa. In addition, there is limited information available on this topic from the rest of the African continent. Furthermore, the study determined whether these websites complied with rules set out by the regulators. From the results, the roles of various stakeholders were outlined with the aim of empowering the consumers to safely purchase medicines online and safeguard consumers against counterfeit medicines.

### Limitations

Websites operated by pharmacies must provide specific policies and procedures for consumers to access such as the delivery and return of medicines. Although this information was initially captured, it was not reported as it was outside the scope of the study to compare the information provided to the regulations and rules. Further research could include analysis of such data.

The researchers neither engaged with the pharmacy owners nor website operators. As most websites consisted of multiple pages, it is possible that information may have been missed. Information was however checked and verified by the second researcher as well the supervisor. Furthermore, while this quantitative study reported the information found on the website further engagement with the pharmacy owner and/or responsible pharmacist could provide further insight into the results received.

### Areas for further research

The study identified multiple stakeholders and further investigation into their perceptions and perceived roles is warranted. For example, there are a number of studies providing insight into consumer motivation to purchase medicines online (Almomani et al. 2023; Bowman et al. 2020; Brijnath, Antoniades & Adams 2015). However, this has not yet been investigated in South Africa.

There are a growing number of pharmacies that use social media platforms to communicate with consumers (Benetoli, Chen & Aslani 2015). The study did not investigate medicines that are sold via social media platforms or pharmacies using social media to advertise their services. Thus, further research into this would give an insight into products and services advertised as well as health information provided.

## Conclusion

The study focused on online pharmacy websites in South Africa, with emphasis on their adherence to regulations. The results from the study showed that the majority of online pharmacies were linked to physical community pharmacies; however, there was a low level of compliance with the minimum standards for pharmacies operating websites, in particular the display of registration details. All pharmacy websites identified in this study as legitimate adhered to regulations requesting patients to submit prescriptions when purchasing Schedule 3–6 medication. In contrast, websites identified as operating illegally, did not provide local contact information and prescriptions were not required for the supply and purchase of medication. The study further identified various stakeholders who could be involved and proposed their roles in safeguarding the online sales of medicines and pharmacy websites.
